# Coaching or gaming? Implications of strategy choice for home based stroke rehabilitation

**DOI:** 10.1186/s12984-016-0127-8

**Published:** 2016-02-27

**Authors:** Mónica S. Cameirão, Asim Smailagic, Guangyao Miao, Dan P. Siewiorek

**Affiliations:** Faculdade das Ciências Exatas e da Engenharia, Universidade da Madeira, Campus Universitário da Penteada, 9020-105 Funchal, Portugal; Madeira Interactive Technologies Institute, Polo Científico e Tecnológico da Madeira, Caminho da Penteada, 9020-105 Funchal, Portugal; Department of Electrical and Computer Engineering, Carnegie Mellon University, Pittsburgh, PA USA

**Keywords:** Stroke, Home-based Rehabilitation, Coaching, Gaming

## Abstract

**Background:**

The enduring aging of the world population and prospective increase of age-related chronic diseases urge the implementation of new models for healthcare delivery. One strategy relies on ICT (Information and Communications Technology) home-based solutions allowing clients to pursue their treatments without institutionalization. Stroke survivors are a particular population that could strongly benefit from such solutions, but is not yet clear what the best approach is for bringing forth an adequate and sustainable usage of home-based rehabilitation systems. Here we explore two possible approaches: coaching and gaming.

**Methods:**

We performed trials with 20 healthy participants and 5 chronic stroke survivors to study and compare execution of an elbow flexion and extension task when performed within a coaching mode that provides encouragement or within a gaming mode. For each mode we analyzed compliance, arm movement kinematics and task scores. In addition, we assessed the usability and acceptance of the proposed modes through a customized self-report questionnaire.

**Results:**

In the healthy participants sample, 13/20 preferred the gaming mode and rated it as being significantly more fun (*p* < .05), but the feedback delivered by the coaching mode was subjectively perceived as being more useful (*p* < .01). In addition, the activity level (number of repetitions and total movement of the end effector) was significantly higher (*p* < .001) during coaching. However, the quality of movements was superior in gaming with a trend towards shorter movement duration (*p* = .074), significantly shorter travel distance (*p* < .001), higher movement efficiency (*p* < .001) and higher performance scores (*p* < .001). Stroke survivors also showed a trend towards higher activity levels in coaching, but with more movement quality during gaming. Finally, both training modes showed overall high acceptance.

**Conclusions:**

Gaming led to higher enjoyment and increased quality in movement execution in healthy participants. However, we observed that game mechanics strongly determined user behavior and limited activity levels. In contrast, coaching generated higher activity levels. Hence, the purpose of treatment and profile of end-users has to be considered when deciding on the most adequate approach for home based stroke rehabilitation.

## Background

Dealing with the social and economical burden resulting from the high number of stroke survivors with permanent disability represents a major challenge for modern societies. The challenge becomes yet higher taking into account the enduring aging of the population worldwide [1] that will consequently result in the increase of the number of individuals with age related diseases such as stroke. For the particular case of the USA, estimates indicate that by 2030, ~4 % of the population will have experienced a stroke, with related costs expected to rise from $71.55 billion to $183.13 billion between 2012 and 2030 [2]. New strategies have to be found to face this upcoming scenario, otherwise it will represent a large burden on healthcare systems and caregivers.

One approach relies on home-based rehabilitation, so that stroke survivors can continue their rehabilitation program after hospital discharge with minimal supervision. Home-based stroke rehabilitation has been increasingly addressed during the last years, and while showing promising results in terms of feasibility and impact on recovery [3, 4] it also poses a number of technical and human challenges. In the concrete case of computer-based rehabilitation, current technology allows offering training scenarios adjusted to the characteristics of users, with detailed progress reports and remote monitorization. Moreover, one of the main advantages relies on the fact that most of these applications have protocols that promote hundreds of task-specific movement repetitions. There is evidence that the conjunction of these two factors, increased number of repetitions and task-specificity, is an important ingredient to achieve reorganization of cortical maps after stroke [5, 6]. Here, technology based solutions can play an important role to increase functional movement practice and impact recovery. There are however challenges when deploying such technologies in the home. One challenge relates to the definition of rehabilitation approaches that are adequate for a home environment. What is the most effective strategy to support users when they have to use these systems on their own or with minimum supervision? Self-managed computerized rehabilitation should be straightforward to use, tailor exercises to the profile of users, address function, set goals, improve self-efficacy, provide instantaneous feedback on performance and be engaging [7–9]. A second challenge in home-based approaches in general is long-term treatment adherence. It has been observed that compliance tends to decrease over time below recommended levels for reasons such as insufficient familiarity with technology, competing commitments, or simply lack of motivation [10–12]. Hence, it is important to investigate what characteristics should be included in such systems so that stroke survivors feel more engaged and motivated to use these tools in a systematic way over long periods of time.

Two paradigms show promise for engaging users and promoting long-term usage of home-based rehabilitation technology: coaching and gaming. The first approach relates to the use of coaching strategies for suggesting exercises, supervising performance, providing appropriate feedback and encouragement for training compliance, and ultimately leading to sustained behavior change. The worth of telephone- or web-based coaching has been shown for encouraging physical activity in overweight adults [13] and cardiac patients [14], walking in persons with Parkison’s Disease [15], or adherence in a treatment of depression [16]. One key aspect of coaching in stroke rehabilitation is the existence of a patient-therapist type of interaction. An association between a positive therapeutic alliance and better treatment outcomes and/or treatment adherence has been reported in neurological rehabilitation [17–19]. Such an alliance has also been observed between clients and relational artificial agents [15, 20] suggesting the feasibility of using virtual coaches as a valid alternative to face-to-face patient-therapist interaction.

The second approach, interactive video gaming for stroke rehabilitation, has enormous potential to motivate and keep patients exercising over longer periods of time. There are several advantages in using games for promoting recovery after stroke. Customized games allow implementing artificial environments for task-specific training that can determine in real-time the most appropriate task parameters for each user based on his/her specific requirements. Hence, games can be adjusted to the individual capabilities of users and realistic goals can be set. Additionally, engagement with the training can be increased by modulating task difficulty in a way that the task is neither too easy nor too hard, while keeping an appropriate balance between the challenge and the required skill set [21]. This means that the higher performing the user the more demanding and challenging the task. Although further clinical evidence is needed, several studies that used rehabilitation paradigms based on the use of serious games have shown better outcomes in stroke patients when compared to standard rehabilitation, with stronger results when customized VR (Virtual Reality) games are used [9, 22, 23]. Some works also suggest the potential of VR games to modulate cortical reorganization after stroke [24–26]. It is however important to take into account that interactive games often pose additional cognitive demands, such as divided attention or dual-tasking, that might interfere with the quality of movements specially in patients with stroke and/or cognitive impairment [27–29]. Hence, while the modulation of cognitive demands has been suggested to be beneficial for enhancing motor learning [30–32], it is mandatory to understand which patients can benefit the most from such approach [33, 34].

Unfortunately, to our knowledge there are no comparative studies investigating the differences in effectiveness, adherence and acceptance between coaching and gaming approaches. This study is a first step in this direction. The goal of this paper is to compare user performance (healthy participants and stroke survivors) of a simple elbow flexion and extension task when the task is performed in a coaching mode (operationally defined in this paper as a mode that provides positive feedback and encouragement) with that in the context of an interactive game. Given the inherent properties of both systems, we hypothesize that 1) enjoyment of training will be superior in the gaming mode leading to increased engagement in the task, and 2) movement execution will have higher quality in the coaching mode because the gaming scenario entails additional cognitive load that may affect movement execution. With this work we aim at gaining new insights on what strategies are more effective when designing technology mediated home based rehabilitation programs, and what system characteristics are needed for their implementation.

## Methods

### Setup

The setup consists of a standard PC with a 17 in. LCD equipped with a Microsoft Kinect 1 (Microsoft, Redmond, WA, USA) for movement tracking. This system uses as basis a Virtual Coach for stroke rehabilitation developed at the Quality of Life Technology Center, Carnegie Mellon University, by Smailagic and co-workers [35]. The Virtual Coach is an intelligent system for encouraging, guiding and monitoring the execution of upper limb exercises that integrates movement tracking, real-time video guidance and emotion recognition to evaluate the physical and emotional state of its users, and that can be integrated in gaming scenarios. For the particular purpose of this study, we developed a modified version of the original system where we just kept the movement tracking, scoring, corrective feedback and gaming features. In addition, we simplified the user interface and developed two different training modes (coaching and gaming, which are described later in this section) that are loosely based on the original version. The system is implemented in C#.NET and uses Microsoft XNA Game Studio 4.0.

### Training modes

**Fig. 1:**
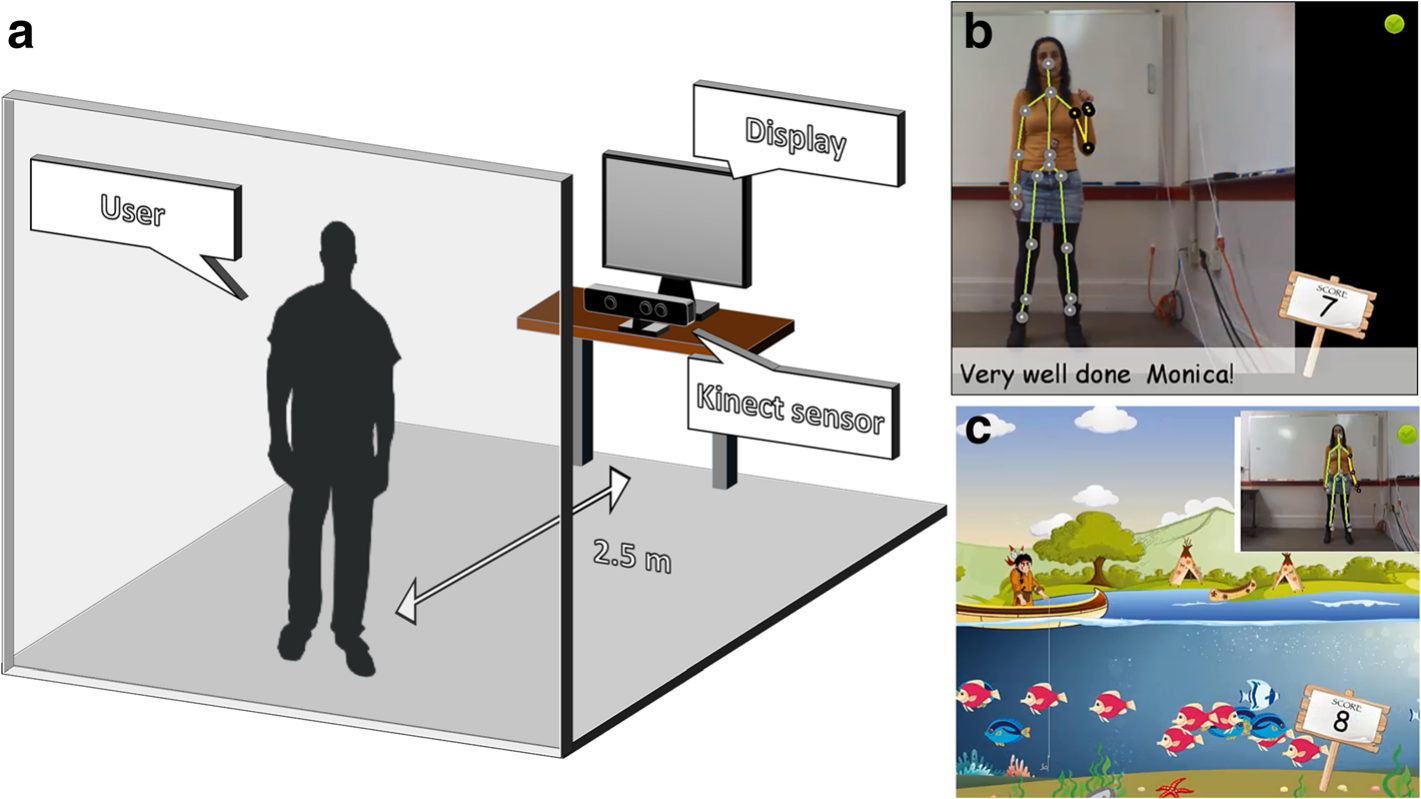
Experimental setup and training modes. **a**) Users perform the exercise while standing and facing a computer screen. Movements are tracked using a Kinect sensor. **b**) Coaching mode: users receive positive and encouraging feedback on each movement sequence. **c**) Gaming mode: the elbow flexion and extension task is mapped to a fishing task within a serious game

While using the system, the user stands facing the computer screen and the Kinect sensor at a distance of approximately 2.5 m, his/her back against a white wall to minimize background noise that could interfere with movement tracking (Fig. 1a). The task consists on performing self-paced elbow flexion and extension. On the screen, the user observes himself/herself performing the task, a training strategy commonly used in rehabilitation where mirror feedback is used for self-correction, movement control and posture retraining [36]. For each movement sequence (starting from full arm extension, executing elbow flexion and back to extension), the user receives a score that ranges from 1 to 10, the later corresponding to excellent performance. In the scoring function, a large range of motion contributes towards a higher score whereas the presence of abduction (compensatory movement) reduces the score. For each individual, the scoring function is calibrated to their maximum range of motion. For the particular purpose of this study, the score was event based and not cumulative to avoid influencing the performance in subsequent conditions. In addition to the score, the user receives audio and written correction cues concerning the alignment of the hand and elbow during the execution of the task. The training can be deployed in two different modes: Coaching and Gaming.

- *Coaching*: mode based on the delivery of positive feedback and encouragement during training. The system has a total of 41 prerecorded feedback sentences, each of them associated to a score range. The categorization of the feedback sentences was done based on the ratings of 10 naive healthy volunteers who were asked to imagine that they were exercising in a gym and that a coach was giving them feedback on their performance. For each feedback sentence, the volunteers rated their perceived performance in a 10-point Likert scale (1 - I performed very poorly; 10 - I performed very well). These ratings were used to allocate the feedback sentences to specific scores. After each movement sequence, and based on the achieved score, audio and written encouragement feedback is given to the user concerning the performance of the movement (Fig. 1b). For example, feedback sentences such as "That was fantastic!" or "That was awesome!" are associated to very high scores (≥8); on the opposite side, feedback sentences such as “Let’s try again. I know you can do better.” Or “That was close!” correspond to lower scores (≤2).
- *Gaming*: mode based on the delivery of feedback through gamified training. In this case, the trained elbow flexion/extension movement previously described is mapped to an avatar performing a virtual fishing task. In this task, the user has to perform elbow flexion and extension to pull and release a fishing line in order to capture the maximum number of fish (Fig. 1c). In this mode, the user receives the same scoring as in the coaching mode but not supported with encouraging feedback sentences.

### Participants

Twenty healthy participants (13 males and 7 females, mean age 35.4 ± 15.1 years) and 5 stroke survivors (4 males and 1 female, mean age 56.6 ± 4.2 years, 4–15 years after stroke) participated in the study after giving their informed consent. Two were African American, 6 Asian and 17 Caucasian. For all participants the inclusion criteria were the following: being at least 20 years of age; being able to read; free-will, cooperation and motivation to participate. Additionally, stroke survivors had remaining deficits in their paretic arm, but with active range of motion against gravity at the elbow. Participants with cognitive and/or vision disorders that could interfere with the understanding, communication and the execution of the task were excluded. The sample was self-selected; all participants enrolled voluntarily through an online participants pool of the Pittsburgh region (Center for Behavioral and Decision Research, Carnegie Mellon University). Participants received a compensation of 20 US Dollars for participation. The study followed standard guidelines for research conducted with human subjects, and was approved by the Carnegie Mellon University Institutional Review Board (IRB protocol number HS13-599).

### Experimental protocol

A within subjects experimental design was used for testing our hypotheses. All participants were exposed to three experimental conditions in which they were instructed to perform elbow flexion and extension movement sequences at their own pace while facing a computer screen: 1) coaching mode (see Coaching in the Training Modes section), in which the user observes himself executing the movements and gets verbal and written encouragement feedback after each movement sequence; 2) gaming mode (see Gaming in the Training Modes section), in which the movements of the user are mapped in realtime onto the movements of an avatar in a game; and 3) control, a condition similar to coaching but without the verbal encouragement. The purpose of the control was to have a condition without the main features of coaching and gaming; hence, all feedback was removed except for the scoring and correction cues that were used throughout the 3 conditions. For all conditions, participants were instructed to perform the movements at a pace that was comfortable for them, to focus on the score after each movement and to be attentive to the suggested correction cues from the software for performance improvement. For the gaming condition, participants received additional explanations on the mechanics of the game. After giving their informed consent, participants underwent a training period with the system until they felt comfortable executing the task. This period was also used to calibrate the task to the maximum range of motion of the user. The 3 conditions were performed in a block, and randomized to control for order effects. Users performed two consecutive blocks. Healthy participants used their dominant arm and stroke survivors used their paretic arm. For healthy users and stroke survivors, each condition had a duration of 6 and 4 min, respectively. After the first block, participants rested and were asked to fill-in the System Usability Scale questionnaire [37]. Stroke survivors additionally answered the Stroke Impact Scale v3.0 [38] as a general assessment on how stroke has affected their life. Participants then proceeded to the second block of conditions. In total, healthy participants and stroke survivors, interacted with the system during 36 and 24 min, respectively, not including the training period. Finally, all participants filled-in a customized self-report questionnaire to assess ease of use, engagement and feedback depending on the training mode (coaching or gaming). This questionnaire was presented in the format of a 5-point Likert scale and participants had to report their agreement/disagreement with respect to a number of statements. All participants also rated on the same scale if they felt tired after the session, independently of the training mode. For stroke survivors, there were additional questions addressing their perception of the potential benefits of the system, and they were also asked about the type of exercises they would like to have should they have the system at their home. Finally, all participants were encouraged to comment and make suggestions on the system.

### Data analysis

We recorded the score obtained in each movement sequence and the time series of the 3D joint positions provided by the Kinect sensor, which allowed us to analyze arm movement kinematics during the experimental sessions. From the raw data we extracted different metrics - that are dependent variables in our experimental design - concerning compliance, movement execution, and performance in the task.

- Compliance: our operational definition of compliance relates to the activity level during the task, which we measure as the *total number of repetitions* (number of elbow flexion and extension sequences executed, independently of being successful or not in achieving a score), and as the *total arm movement* during training computed as the amount of movement of the end effector during the entire session.
- Movement execution: for each experimental condition we computed: 1) the median *range of motion*, computed as the absolute linear distance between start and end positions of the hand in the elbow flexion and extension sequence 2) the median *movement duration*, 3) the median *travel distance* computed as the length of the trajectory between start and end positions in the elbow flexion and extension sequence, 4) the interquartile range (IQR) of all the previous metrics as a measure of *variability*, and 5) *movement efficiency* computed as the ratio between the range of motion and the travel distance.
- Performance: we used the median *score* of all movement sequences and its IQR as a measure of overall performance during training.

For each dependent variable, the normality of the distribution was assessed using the Kolmogorov-Smirnov normality test. Because the data deviated from normality, non-parametric statistical tests were used for the analysis. For the assessment of overall differences between the three experimental conditions, a Friedman test was used on each dependent variable. For further pairwise comparisons, the Wilcoxon's T matched pairs signed ranks test was used. This same test was used for analyzing the self-reported data, because of the ordinal nature of the questionnaire, and comparing the coaching and gaming modes. For all pairwise comparisons, a Bonferroni correction was used to account for the number of comparisons. Effect sizes were computed on the pairwise comparisons. For all statistical comparisons the significance level was set to α = .05. All statistical analysis was done using IBM SPSS 22.0 (SPSS Inc., Chicago, IL, USA).

For stroke survivors, only a descriptive analysis was done because of the small sample size (*N* = 5).

## Results

### Results for healthy participants

See Table 1 for a concise summary of the central tendency for compliance, movement execution and performance metrics during the experimental conditions.

**Table 1:** Summary of metrics within the Compliance, Movement Execution and Performance domains for healthy participants for the three experimental conditions

Variable	Control	Coaching	Gaming	*p*-value
Compliance				
Nr Repetitions	**171.5 (54)**	**177.0 (52)**	**91.5 (9)**	**<0.001**
Total Movement (m)	**171.9 (43.8)**	**170.1 (61.1)**	**91.0 (35.7)**	**<0.001**
Movement Execution				
Duration (s)	1.67 (0.47)	1.44 (0.55)	1.24 (0.63)	0.074
Duration Variability (s)	**0.60 (0.24)**	**0.53 (0.25)**	**0.81 (0.33)**	**0.001**
Range of Motion (m)	0.69 (0.11)	0.68 (0.15)	0.67 (0.13)	0.387
Range of Motion Variability (m)	0.04 (0.02)	0.04 (0.04)	0.04 (0.02)	0.387
Travel Distance (m)	**0.92 (0.21)**	**0.93 (0.27)**	**0.84 (0.19)**	**<0.001**
Travel Distance Variability (m)	**0.13 (0.09)**	**0.12 (0.10)**	**0.09 (0.07)**	**0.022**
Movement Efficiency	**0.76 (0.06)**	**0.77 (0.09)**	**0.83 (0.07)**	**<0.001**
Performance				
Score	**8.1 (1.3)**	**8.0 (1.1)**	**8.5 (1.1)**	**<0.001**
Score Variability	**0.9 (0.5)**	**0.8 (0.6)**	**0.5 (0.4)**	**<0.001**

The values are represented as median (IQR), together with the probability values resulting from the Friedman test. Bold values indicate a significant effect

#### Does gaming increase compliance?

**Fig. 2:**
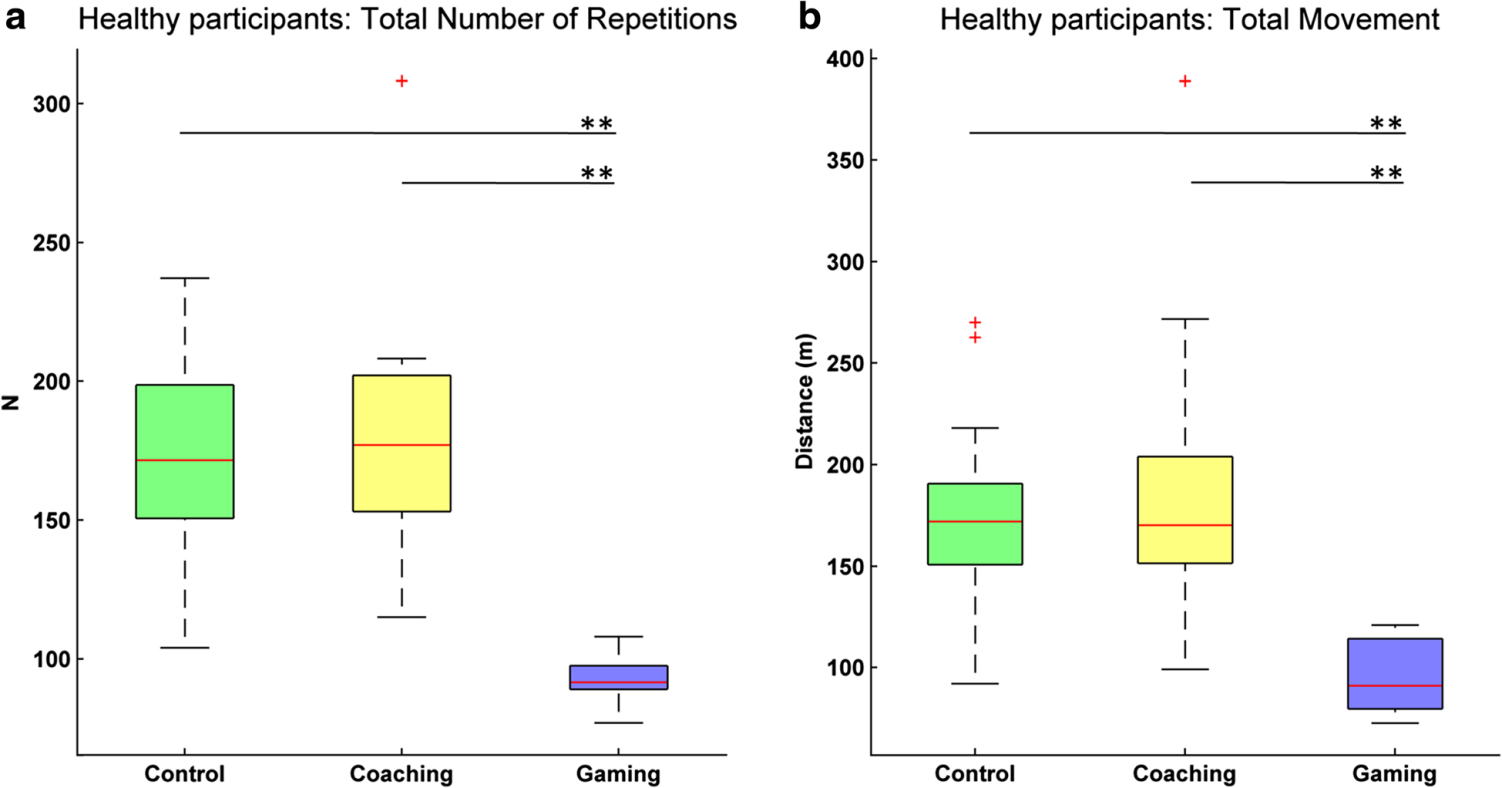
Compliance in the three experimental conditions for healthy participants. **a**) The total number of repetitions (number of elbow flexion and extension sequences) is significantly lower during the gaming mode. **b**) The same trend is observed for the total amount of movement of the end effector during the training session. ** *p* < .001

Our first hypothesis stated that the gaming mode would be more enjoyable and lead to increased compliance during training, as measured by the number of exercise repetitions and the total arm movement during training, when compared to the coaching mode. The median of the total number of repetitions per participant was 171.5, 177.0 and 91.5 for control, coaching and gaming conditions, respectively. The median of the total arm movement per participant was 171.9, 170.1 and 91.0 m for control, coaching and gaming, respectively. A non-parametric repeated measures analysis showed that differences across conditions were significant for the number of repetitions (Fr (2) = 30.10, *p* < 0.001) and also for the total arm movement during training (Fr (2) = 30.40, *p* < 0.001). Further pairwise comparisons revealed that the total number of repetitions during gaming were significantly less than during coaching (*T* = 0, *p* < 0.001, *r* = −0.62) and during control (*T* = 0, *p* < 0.001, *r* = −0.62); no significant difference was found between control and coaching (*T* = 78.5, *p* = 0.32, *r* = −0.16) (Fig. 2a). The same trend was observed for the total activity during training with participants showing significantly less movement during gaming when compared to coaching (*T* = 0, *p* < 0.001, *r* = −0.62) and control (*T* = 0, *p* < 0.001, *r* = −0.62); again, no significant difference was found between control and coaching (*T* = 74, *p* = 0.25, *r* = −0.18) (Fig. 2b).

#### Is the quality of movements inferior during gaming?

Our second hypothesis stated that the quality of the movements, as measured by the duration, length and efficiency of movements, would be inferior in the gaming mode when compared to the coaching mode.

**Fig. 3:**
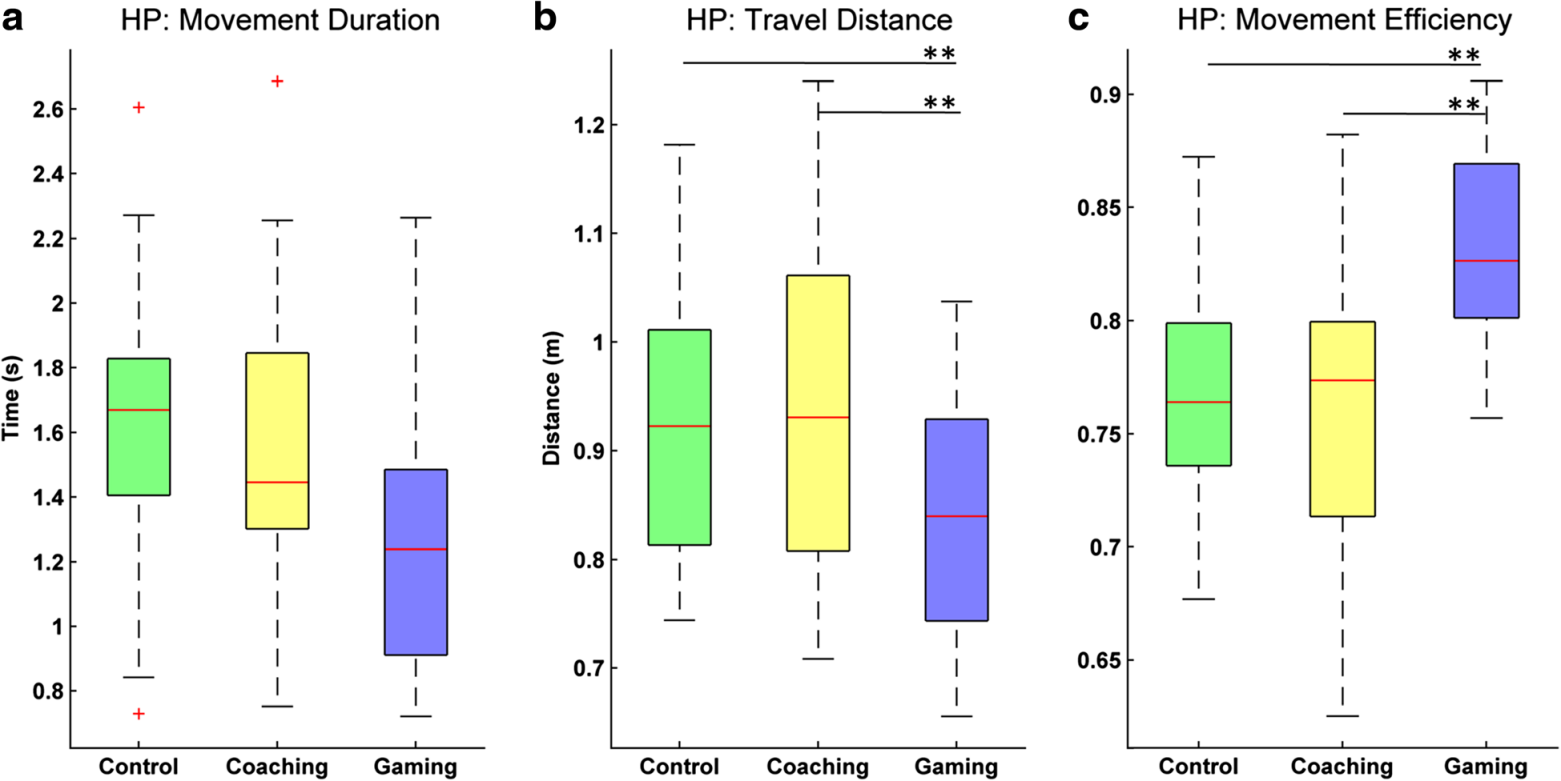
Movement execution metrics in the three experimental conditions for healthy participants (HP). **a**) Duration of movements; **b**) Median travel distance computed as the length of the trajectory between start and end positions; and **c**) Movement efficiency computed as the ratio between the range of motion and the travel distance. ** *p* < .001

*Duration of movements*: the duration of movements tended to be shorter during the gaming mode, but differences across conditions were not significant (Fig. 3a). However, there was a significant difference for the variability in movement duration (Fr (2) = 13.30, *p* < 0.01). Specifically, pairwise comparisons showed that the movements during gaming had significantly more variability in time duration when compared to coaching (*T* = 14.0, *p* < 0.01, *r* = −0.54) and control (*T* = 29.0, *p* < 0.01, *r* = −0.45). There were no significant differences between coaching and control conditions.

*Length*: differences across conditions were significant for the median travel distance (Fr (2) = 21.70, *p* < 0.001) and its variability (Fr (2) = 7.60, *p* < 0.05). Pairwise comparisons showed that the travel distance was significantly shorter for the gaming condition when compared to coaching (*T* = 5.0, *p* < 0.001, *r* = −0.59) and control (*T* = 1.0, *p* < 0.001, *r* = −0.61) (Fig. 3b). In addition, the variability in the travel distance during gaming was significantly lower when compared to coaching (*T* = 38.0, *p* < 0.016, *r* = −0.40) and control (*T* = 33.0, *p* < 0.016, *r* = −0.43). Pairwise comparisons for the previous metrics did not show significant differences between coaching and control conditions. Finally, the range of motion was similar across conditions, and also its variability, with no significant effect across conditions.

**Fig. 4:**
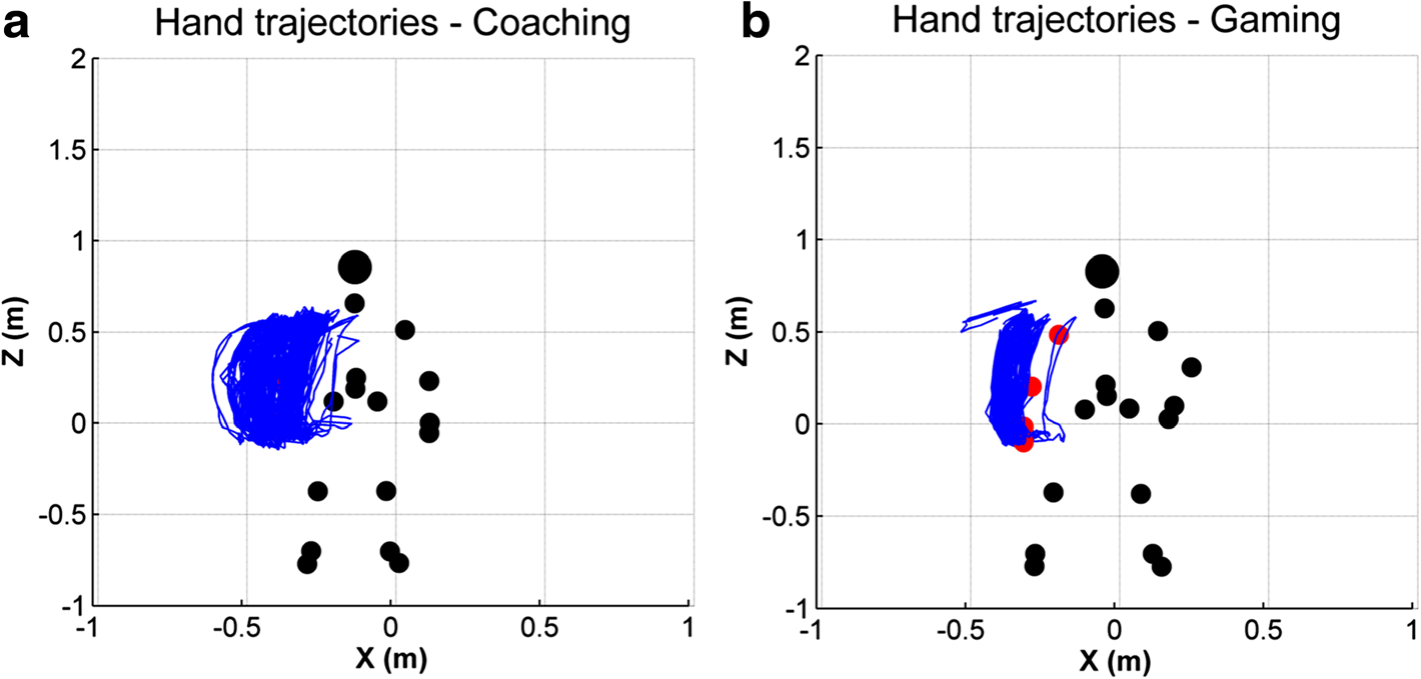
Hand trajectories during training. The trajectories shown were recorded over one block of **a**) coaching and **b**) gaming for a 45 years old female. The figures show a front view of the participant, who performed the exercises with her right arm

*Efficiency*: differences across conditions were significant for the movement efficiency (Fr (2) = 30.0, *p* < 0.001), computed as the ratio between the range of motion and the travel distance. Specifically, efficiency was significantly higher during the gaming condition when compared to coaching (*T* = 0.0, *p* < 0.001, *r* = −0.62) and control (*T* = 0.0, *p* < 0.001, *r* = −0.62) (Fig. 3c). This difference in efficiency can be observed when comparing examples of hand trajectories in coaching and gaming conditions. Figure 4 shows one such example for a healthy participant, where we can observe that trajectories are less dispersed during gaming. There were no significant differences between coaching and control conditions.

#### Is performance modulated by the training mode?

**Fig. 5:**
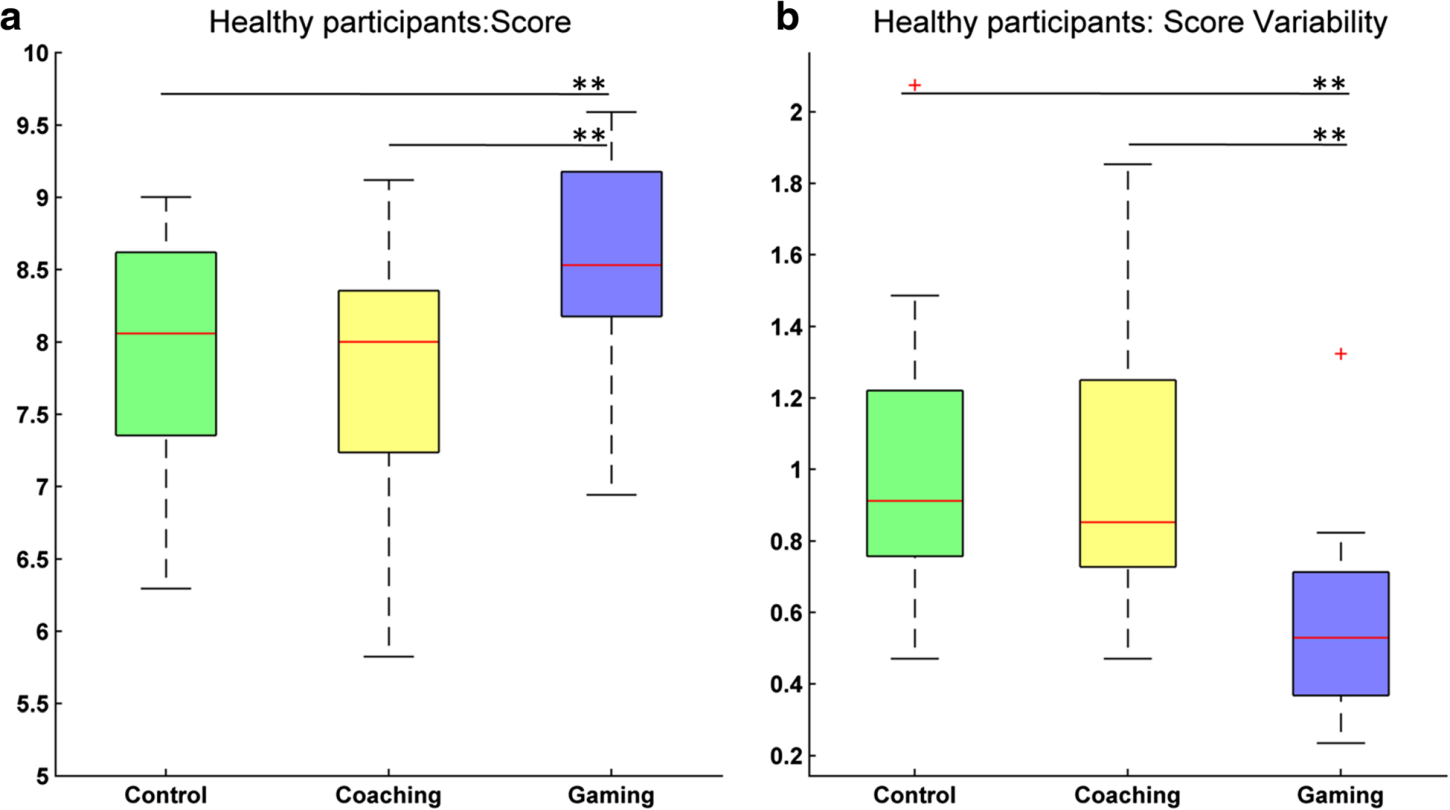
Performance in the three experimental conditions for healthy participants. **a**) Median score per participant over all elbow flexion and extension movement sequences; and **b**) Variability (IQR) in score. ** *p* < .001

For analyzing the overall performance in the different experimental conditions, we extracted the median score per participant and score variability over all repetitions. We found significant differences across conditions for both the score (Fr (2) = 25.0, *p* < 0.001) and its variability (Fr (2) = 28.545, *p* < 0.001). We registered a median score of 8.1, 8.0 and 8.5 for control, coaching and gaming, respectively. Pairwise comparisons revealed that the score during gaming was significantly higher when compared to coaching (*T* = 1.5, *p* < 0.001, *r* = −0.61) and control (*T* = 3.5, *p* < 0.001, *r* = −0.58) (Fig. 5A). In addition, the variability during gaming was significantly lower than during coaching (*T* = 0.0, *p* < 0.001, *r* = −0.62) and control (*T* = 4.0, *p* < 0.001, *r* = −0.60) (Fig. 4B). No differences were found between coaching and control conditions.

### Usability and acceptance

Overall, the systems used during this study were rated as having very good usability, with an average score of 84.6 ± 13.5 in the System Usability Scale. When asked about which training mode they preferred, 65 % of the participants selected the gaming mode while 35 % preferred the coaching mode. Analyzing the ratings of individual questions of the customized self-report questionnaire (Table 2), we observed that there were no significant differences in the ratings between the coaching and gaming modes, except for two aspects: feedback (Q6) and enjoyment (Q10). Overall, the median ratings were positive for both modes concerning ease of use (Q3, Q4 and Q9), engagement (Q1, Q10, Q11 and Q12) and the delivered feedback (Q2, Q5, Q6, Q7 and Q8). Concerning the feedback component (Q6), participants considered that the feedback provided in the coaching mode was significantly more useful than that provided in the gaming mode (*T* = 6.0, *p* = 0.003, *r* = −0.47). Regarding enjoyment (Q10), users rated the gaming mode as being significantly more fun than the coaching mode (*T* = 0.0, *p* = 0.034, *r* = −0.34). Finally, on average participants reported that they felt tired after the session (Mdn = 4.0).

**Table 2:** Median ratings per statement in the self-report questionnaire for healthy participants

Question	Coaching	Gaming	*p*-value
**Q1.** Exercising with the Virtual Coach was entertaining.	4.0 (1.0)	4.0 (1.0)	0.053
**Q2.** I found that the dialogue/interaction with the Virtual Coach was natural.	3.0 (2.0)	4.0 (2.0)	0.293
**Q3.** The task was easy to understand.	5.0 (0.0)	5.0 (0.0)	0.317
**Q4.** The exercise was easy to execute.	5.0 (1.0)	5.0 (1.0)	1.000
**Q5.** It was difficult to understand how well I was doing the exercises.	1.0 (1.0)	1.0 (1.0)	0.655
**Q6.** The feedback provided by the Virtual Coach was useful to me.	**5.0 (1.0)**	**3.0 (2.0)**	**0.003**
**Q7.** The score was difficult to understand.	2.0 (2.0)	2.0 (1.0)	0.680
**Q8.** When I did not perform correctly, it was easy to understand what I did wrong.	4.0 (3.0)	4.0 (2.0)	0.161
**Q9.** The training was easy.	5.0 (1.0)	5.0 (1.0)	1.000
**Q10.** The training was fun.	**3.5 (1.0)**	**4.0 (2.0)**	**0.034**
**Q11.** I felt that through voice, the software kept me engaged with the exercises.	4.0 (2.0)	4.0 (2.0)	0.527
**Q12.** I found the encouragement after each repetition useful.	4.0 (2.0)	--	--

Bold values indicate a significant effect

### Results for stroke survivors

The stroke survivors that participated in the study presented different levels of chronic physical impairment derived from stroke as assessed by the Physical Domain (includes strength, hand function, mobility and ADL/IADL) of the Stroke Impact Scale (Table 3).

**Table 3:** Demographic information of stroke survivors. In the Stroke Impact Scale, each domain has a maximum score of 100. The Physical Domain encompasses strength, hand function, mobility, and ADL/IADL

Variable	S1	S2	S3	S4	S5
Demographics					
Age	59	62	52	57	53
Gender (M/F)	M	M	M	F	M
Months post stroke	175	113	114	183	58
Stroke type (Ischemic/Hemorrhagic)	H	H	I	-	H
Paretic Arm (L/R)	L	R	L	R	L
Stroke Impact Scale					
Physical Domain	79.7	96.9	40.2	94.6	60.6
Memory	82.1	100	67.9	89.3	50.0
Communication	89.3	100	92.9	96.4	64.3
Emotion	80.6	97.2	58.3	91.7	61.1
Handicap	81.3	93.8	75.0	96.9	50.0
Stroke Recovery	70	95	65	95	75

The results per participants and the central tendency for compliance, movement execution and performance metrics can be found in Table 4. Concerning compliance during training, we observed the same trend as with healthy users. The median total number of repetitions during gaming were less than during coaching and control, and these were similar during coaching and control. Likewise, the median total activity during training was less during gaming than during coaching and control. These findings are also preserved when analyzing the participants individually, except for participant S5.

**Table 4:** Compliance, Movement Execution and Performance metrics for stroke survivors for the 3 experimental conditions

Variable	S1	S2	S3	S4	S5	Med (IQR)		
						Control	Coaching	Gaming
Compliance								
Nr Repetitions	108/91/72	120/126/58	110/125/47	115/119/54	135/159/158	115.0 (19)	125.0 (38)	58.0 (65)
Total Movement (m)	194.7/158.7/93.0	154.4/154.6/74.6	36.2/39.2/19.8	100.5/98.8/60.8	101.7/106.1/135.1	101.7 (106.2)	106.1 (87.6)	74.6 (73.7)
Movement Execution								
Duration (s)	1.10/1.09/1.17	1.74/2.01/1.61	1.60/1.43/1.70	1.84/1.83/1.72	1.94/1.80/1.82	1.74 (0.54)	1.80 (0.66)	1.70 (0.38)
Duration Variability (s)	0.56/0.99/0.32	0.75/0.67/2.85	0.71/0.80/0.49	0.66/0.44/1.34	0.28/0.29/0.80	0.66 (0.31)	0.67 (0.53)	0.80 (1.69)
Range of Motion (m)	0.96/0.91/0.76	0.84/0.80/0.83	0.17/0.14/0.15	0.67/0.65/0.69	0.62/0.60/0.67	0.67 (0.50)	0.65 (0.49)	0.69 (0.38)
Range of Motion Variability (m)	0.07/0.05/0.07	0.05/0.03/0.03	0.06/0.05/0.04	0.05/0.08/0.06	0.04/0.06/0.09	0.05 (0.02)	0.05 (0.02)	0.06 (0.04)
Travel Distance (m)	1.27/1.25/0.99	1.21/1.16/1.12	0.26/0.23/0.19	0.88/0.89/0.92	0.77/0.76/0.83	0.88 (0.73)	0.89 (0.71)	0.92 (0.55)
Travel Distance Variability (m)	0.40/0.30/0.18	0.20/0.15/0.14	0.18/0.20/0.11	0.10/0.11/0.11	0.08/0.06/0.17	0.18 (0.21)	0.15 (0.16)	0.14 (0.07)
Move Efficiency	0.75/0.73/0.77	0.70/0.69/0.74	0.66/0.59/0.79	0.76/0.74/0.75	0.81/0.79/0.81	0.75 (0.10)	0.73 (0.13)	0.77 (0.06)
Performance								
Score	6.9/6.4/7.0	8.6/8.3/8.4	7.2/7.6/8.0	7.8/8.1/7.9	8.4/6.3/8.1	7.8 (1.5)	7.6 (1.8)	8.0 (0.8)
Score Variability	1.0/0.9/1.1	0.6/0.8/0.8	1.3/1.2/0.5	0.9/0.6/0.6	1.5/1.6/1.6	1.0 (0.7)	0.9 (0.7)	0.8 (0.8)

In terms of movement execution, for the duration of movements there is a trend towards executing faster movements during gaming, but not so evident as with healthy participants. As observed also in healthy participants, the variability in the duration of movements was higher during gaming. Individually, participants S2, S4 and S5 displayed this pattern. Participants S1 and S3 showed faster movements and more variability in the duration of movements during the coaching condition. On what concerns the median range of motion, it was mostly unaffected across conditions, as observed also in healthy participants. Participant S1 only displayed a smaller range of motion during gaming. Regarding the median travel distance during the training sessions, this was alike across conditions. This is not in accordance with what we observed for healthy participants, who showed a significantly shorter travel distance during gaming. At the individual level, S1 only showed a smaller distance during gaming. This is in accordance with the smaller range of motion of this participant during that condition. Finally, the median efficiency of movements tended to be higher during gaming, as observed for healthy participants. Most stroke survivors showed this trend except for participants S4 and S5 who showed similar values of efficiency for the 3 conditions. For the overall performance, measured as the median score over all movement sequences, we did not find a clear trend. Participants S1 and S3 scored higher during the gaming condition, while participants S2 and S5 scored higher in the control condition; and participant S4 scored higher during coaching.

Regarding usability, stroke participants rated the system as being very usable with an average score of 86.5 ± 12.3 in the System Usability Scale questionnaire. In terms of mode preference, participants S2, S3 and S5 preferred the gaming mode, while participants S1 and S4 preferred the coaching mode. Analyzing the answers to the questions of the customized self-report questionnaire (Table 5), stroke participants considered that the system was easy to use in both its modes (Q3, Q4 and Q9). Concerning engagement, ratings were similar for both modes (Q1, Q10, Q11 and Q12) but the participants rated the gaming mode as being more entertaining (Q1). When asked about the feedback delivered by the system, stroke survivors rated both modes similarly (Q2, Q5, Q6, Q7 and Q8). Additionally, stroke users reported that they thought that the system (despite its mode) would help them improve their movements (Q14) and performance in activities of daily living (Q15). Lastly, when participants were asked if they felt tired after the session, the median rating was 3.0.

**Table 5:** Median ratings per statement in the self-report questionnaire for stroke survivors

Question	Coaching	Gaming
**Q1.** Exercising with the Virtual Coach was entertaining.	3.0 (1.0)	5.0 (1.0)
**Q2.** I found that the dialogue/interaction with the Virtual Coach was natural.	4.0 (2.0)	4.0 (2.0)
**Q3.** The task was easy to understand.	4.0 (2.0)	4.0 (2.0)
**Q4.** The exercise was easy to execute.	5.0 (2.0)	4.0 (2.0)
**Q5.** It was difficult to understand how well I was doing the exercises.	2.0 (1.0)	2.0 (1.0)
**Q6.** The feedback provided by the Virtual Coach was useful to me.	4.0 (1.0)	4.0 (1.0)
**Q7.** The score was difficult to understand.	2.0 (1.0)	2.0 (2.0)
**Q8.** When I did not perform correctly, it was easy to understand what I did wrong.	3.0 (1.0)	3.0 (3.0)
**Q9.** The training was easy.	4.0 (2.0)	4.0 (2.0)
**Q10.** The training was fun.	5.0 (2.0)	5.0 (1.0)
**Q11.** I felt that through voice, the software kept me engaged with the exercises.	4.0 (1.0)	4.0 (2.0)
**Q12.** I found the encouragement after each repetition useful.	4.0 (2.0)	--
**Q14.** I think this system would help me improve my movements.	4.0 (1.0)	4.0 (1.0)
**Q15.** I think this system would help me improve my performance in activities of daily living.	4.0 (2.0)	4.0 (2.0)

When asked about the type of exercises they would like to do should they have this system at home, stroke survivors tended to suggest exercises related to their particular perceived limitations. For example, participant S1 would like to have *“… Fine (small) operations (movements)*”, while S2 referred “E*xercises related to my left leg (hip, knee, angle). Movement and strength.*”, and S4 mentioned “*Exercises with fingers. Exercises with foot and toes - maybe plantar flexion and dorsiflexion, and then rotation of foot/toes in each position…*”. Stroke survivors also suggested exercises related to their personal preferences. For example, participant S3 would like to have “*more game based activities …*”, while S4 suggested “*… doing it as part of a tai chi game*.” and S5 advocated for “*baseball and basketball*” exercises.

## Discussion

When dealing with home-based self-managed rehabilitation, one of the main problems is the use rate decrease over time for reasons such as frustration in the use of the affected limb, lack of motivation to exercise, or simply difficulty in implementing a systematic training routine [10, 12]. Coaching and gaming arise as promising approaches for increasing engagement, the first through supervision and delivery of supportive feedback, and the second by adapted game play. We investigated performance of an elbow flexion and extension task within two training modes that recreated in a very simple way coaching and gaming paradigms. Coaching was based on the delivery of supportive encouragement and gaming on performing the very same task within a custom video game. The training modes were deliberately simplified to rule out confounding factors that could hamper the interpretation of the results. Table 6 provides a general overview of the main results when comparing coaching and gaming modes in the healthy participants sample.

**Table 6:** Overview of main results for coaching and gaming modes. The table reflects the results of significant pairwise comparisons between coaching and gaming modes for healthy participants

	Coaching vs Gaming
Number of repetitions	Coaching > Gaming
Total movement	Coaching > Gaming
Duration of movements	Coaching > Gaming
Travel distance	Coaching > Gaming
Movement efficiency	Gaming > Coaching
Score	Gaming > Coaching
Enjoyment	Gaming > Coaching
Perceived usefulness	Coaching > Gaming

We hypothesized that enjoyment would be higher in the gaming mode and that this would translate to more activity, i.e., more movement and an increased number of task sequences during training. When asked about the preferred training mode 65 % of the healthy participants selected the gaming mode, indicating that gaming was considered more enjoyable, independently of age and gender for this particular population. This was corroborated by a significantly higher rating in gaming when healthy participants subjectively rated how fun the training was. The same trend has been observed in the literature when comparing game based rehabilitation to other approaches [12, 39]. However, contrary to our prediction, the activity level of healthy participants during gaming was significantly lower than during the coaching mode and stroke survivors showed a similar trend. We observed less movement of the end effector and less repetitions. Specifically for repetitions, the median number over the 12 min of each condition for healthy subjects was 91.5 for gaming and 177.0 for coaching. We registered 58.0 and 125.0 for gaming and coaching respectively, over 8 min for stroke survivors. Peters et al. [40] observed a related effect when comparing lower extremity stepping during video game to that performed in traditional rehabilitation. The authors observed fewer repetitions during the video game sessions because the game required the users to adopt a standing position without support in front of the monitor that may have limited their performance. In our case, the dynamics of the selected game required a fish to bite the hook in order to achieve a successful movement sequence, what added more pause between sequences in gaming than in the coaching mode. In fact, this is not unique to our game. The temporal dynamics of gameplay are strongly influenced by the game mechanics, which determine the pace at which a game can be played. However, this limitation does not apply to coaching approaches because these are fully self-paced and not constrained by game mechanics. This is an important aspect that has to be critically addressed when developing solutions for stroke rehabilitation, particularly for patients in the acute phase. Animal studies suggest that hundreds of active functional movement repetitions per day could be required for cortical plasticity; brain reorganization was observed in rats and squirrel monkeys after 400–600 skilled movement repetitions per day [41, 42]. In humans, stroke survivors that performed protocols with hundreds of movement repetitions displayed cortical plasticity and better functional improvements than controls with lower doses of treatment [43, 44]. Hence, any rehabilitation program should promote the execution of an adequate number of movement repetitions to optimize recovery. Unfortunately, current movement practice during stroke rehabilitation tends to be insufficient [45, 46]. For example, Lang et al. observed 312 conventional therapy stroke rehabilitation sessions and reported that on an average 36 min session, patients executed 54 active movement sequences of the upper extremity [45]. Extrapolating our results from 5 stroke survivors to a 36 min session and assuming one fourth of rest period, it would be possible to achieve about 196 repetitions during gaming and 422 repetitions during coaching, with coaching being more than two times more efficient than gaming in terms of repetitions. This means that we could reach satisfactory movement practice with the coaching mode, but would fall short with the current interactive game, although reaching much better activity levels than those reported in the literature for conventional therapy. Nevertheless, dosing depends on three key parameters: (1) training duration, (2) frequency with which the individual performs training, and (3) number of repetitions performed during training. Consequently, the reduced number of repetitions found in gaming could be compensated through longer training duration or increased frequency of sessions through distributed training. There is however still a debate on how to combine training duration, number of repetitions, and frequency, and their specific impact on recovery after stroke, with the more consensual idea being that larger doses of therapy lead to improved outcomes [5, 47]. On the other hand, animal studies suggest that the mere repetition of movements involving little or no learning, does not induce plastic changes in motor maps [6, 48]. For this reason, rehabilitation training should be task-specific and always pose motor challenges for post-stroke subjects [5, 49]. In this sense, a gaming approach could have a larger potential because it can generate challenges of increasing difficulty adapted to each patients' capabilities. Unfortunately, there is no general solution for adapting difficulty levels during gaming. Such a process requires to study the psychometrics of the game with end-users in order to provide personalized gaming parameters adjusted to the capabilities of each user [50], which is not only highly demanding but also game specific.

Concerning the executed movements, we assumed that the gaming mode would entail higher cognitive load because it requires the execution of elbow and extension movements while controlling also the fishing timing. Hence, we expected that it would result in a decrease of quality in the executed movements [27–29]. We observed for healthy participants that during gaming the elbow flexion and extension sequences tended to be faster but with significantly more variability. This could indicate that movements are more ballistic although executed with less temporal precision during gaming. In addition, the travel distance of the end effector was significantly smaller and with significantly less variability. Interestingly, all this evidence suggests that during gaming there is a better performance of movements because movements are faster, and trajectories are more efficient and repeatable as opposed to what was expected. Although there was large individual variability among stroke survivors, the overall trend is also consistent with what was observed for healthy participants. This observation has however to be interpreted with caution considering the small sample size of stroke survivors. This increased quality of the movement during gaming is in accordance with higher movement performance scores as computed by the system, both for healthy participants and stroke survivors. Our interpretation for this result is that during gaming there is an external focus of attention, which has been reported to lead to superior movement quality when compared to tasks that are more focused on the patterns of movements, as is the case of the coaching mode [51]. Our results with healthy participants are in accordance with kinematic studies that have observed that movement execution was significantly shorter in time in tasks with feedback on movement effects (external focus of attention) when compared to tasks with feedback on body movement, both in healthy participants [52] and stroke survivors [53]. We did however not observe this effect in stroke survivors S1, S3 and S5, with movement duration being unaffected or higher in gaming when compared to coaching. Interestingly, these participants were the ones with more physical impairment as measured by the physical domain of the Stroke Impact Scale (79.7, 40.2 and 60.6 over 100 for S1, S3 and S5, respectively). Although our sample is insufficient for making definite claims, we speculate that this could suggest that stroke survivors with higher levels of impairment may not benefit from tasks with an external focus of attention as much as users with less impairment. This interpretation is consistent with studies that have observed that the effects of attention focus are dependent on the skill and impairment level of the users [54–56].

It was interesting to observe in healthy participants that although gaming led to fewer repetitions, a higher quality of movements was achieved. This raises interesting questions concerning the relationship between quantity and quality, and also how dosing, challenge and task-specificity are factors that may influence them. Are less repetitions of better quality more efficient? However, a movement may have a different quality depending on different criteria. A movement may be accurate but not smooth, or quick but not controlled. A similar problem is observed with the concept of quantity or dose in stroke rehabilitation, being it ill-defined [5]. It is imperative to develop unified definitions and metrics that allow for objective comparisons between clinical studies that evaluate the impact of the above mentioned factors in recovery after stroke.

In terms of usability, the system (mode unspecific) was rated with an average usability above approximately 85 over 100 (84.6 and 86.5 in the System Usability Scale for healthy participants and stroke survivors, respectively) which can be considered very good taking into account that scores above 68 in the System Usability Scale questionnaire are considered to be above average [57]. Additionally, both training modes were considered easy to use. However, healthy participants considered the feedback delivered in the coaching mode to be significantly more useful than the one delivered in the gaming mode. Interestingly, the provided feedback on performance (corrective cues and score) was the same in both modes, except for the supportive statements in the coaching mode which provided encouragement accordingly to the achieved score in each movement sequence. Finally, when stroke survivors were asked about particular exercises they would like to perform with such system, there was a trend towards selecting exercises directed to their particular perceived deficits and personal preferences (tai-chi, baseball, etc. …). This suggests that for increasing usage and adherence in home based rehabilitation systems, tasks should be individualized and directed towards the specific perceived deficits and needs of each user, and, when possible, in the context of activities that stroke survivors enjoy.

The results of this preliminary study allowed us to observe that choices on specific features of computer based rehabilitation approaches should be carefully weighed depending on the profile and goal of end-users. Coaching is thought to provide support strategies to help achieving an internally driven behavioral change, whereas gaming, as shown in our results, can be used as an external driver. Hence, this does not mean that therapists and patients necessarily have to choose one over the other. In fact, we believe that an optimal approach would be one that extracts the most beneficial features of these approaches and combines them into a single paradigm. Such a broader approach could then be customized for targeting patients with different profiles, needs and preferences.

In summary, a number of aspects should be considered when developing home-based stroke rehabilitation solutions based on coaching or gaming. Although gaming has shown key benefits for training engagement and quality in comparison to coaching, these might come at the expense of insufficient movement practice if gaming scenarios pose excessive constraints. We believe that decisions on the adequateness of each approach should be based on the end-goal of treatment and stroke stage of the target user. Stroke patients in an acute stage will in principle benefit more from a solution that promotes more repetitions that can lead to faster to higher levels of recovery. Hence, we speculate that a coaching approach would be more adequate. However, for long-term home based treatment past the acute stage of stroke aimed at fitness and maintenance, a gaming approach could be more adequate and lead to more assiduous training. Finally, for our gaming condition, movement execution tended to be more ballistic and with less control. Hence, if the end goal of treatment is fine motor execution training, a coaching approach would be probably more appropriate. Regardless of the selected approach, any solution should be directed towards training the specific perceived functional limitations of each user in the context of activities of personal preference.

This study intended to explore the specificities of coaching and gaming approaches and their benefits and drawbacks as tools to support home training. However, there are some limitations that should be taken into account. First, the sample size is limited, particularly the sample of stroke survivors, what limits the interpretation of results in the context of end users. Second, we believe that the results for the control condition were not significantly different from the results for the coaching condition because these two conditions were not different enough to disclose any effect. We think that a condition in which all feedback (correction cues and score) is removed would have been a more adequate control condition. Third, participants felt tired during the session. Particularly for stroke survivors, this might have generated additional variability in the data because the duration of training was not adjusted to the individual impairment levels of the users.

## Conclusions

In this paper we compared two promising approaches for home-based stroke rehabilitation, coaching and gaming, and aimed at identifying key characteristics of each mode that should be taken into account for future development and deployment. For healthy participants, the gaming mode was considered more enjoyable, a key factor for improving treatment adherence. However, the activity level was affected by the game dynamics, and hence participants were more active during the coaching mode because it was fully self-paced. Stroke survivors showed a similar trend. Data on movement execution during gaming on healthy participants has however been shown to lead to increased movement quality, possibly because the focus of attention is on movement effects and not on movement patterns. Yet, this might be influenced by the impairment level when used by patients. Finally, both training modes have shown high acceptance in both healthy participants and stroke survivors, although healthy participants rated significantly higher the perceived usefulness of the feedback in coaching.

We showed that the choice of the training paradigm in computer based approaches influences performance and we discussed the potential implications for stroke rehabilitation. As follow-up work, it is now important to evaluate the specific performance effects of gaming and coaching in a large sample of stroke survivors, and also the impact of recovery through a controlled clinical trial. Further, an assessment at the home of participants is required to evaluate long term use and adherence.
